# 3D Paper-based milk adulteration detection device

**DOI:** 10.1038/s41598-022-17851-3

**Published:** 2022-08-11

**Authors:** Subhashis Patari, Priyankan Datta, Pallab Sinha Mahapatra

**Affiliations:** Micro Nano Bio-Fluids Group, Department of Mechanical Engineering, Indian Institute of Technology Madras, Chennai, 600036 India

**Keywords:** Chemical engineering, Mechanical engineering

## Abstract

Milk adulteration is a common problem in developing countries, and it can lead to fatal diseases in humans. Despite several studies to identify different adulterants in milk samples, the effects of multiple adulterants remain unexplored. In this work, a three-dimensional (3D) paper-based microfluidic device is designed and fabricated to simultaneously detect multiple chemical adulterants in milk. This device comprises a top cover, a bottom cover, and a middle layer composed of transportation and a detection zone. By making cuts on the middle layer’s support, the device’s flow path is characterised by optimum and uniform velocity. For the first time, seven adulterants (urea, detergents, soap, starch, hydrogen peroxide, sodium-hydrogen-carbonate, and salt) are detected in the milk sample simultaneously with specificity evaluation and detailed color interference analysis. Only 1–2 mL of sample volume is required to detect 7 adulterants at one time. We have used only 10 $\upmu$L of the reagent’s volume for the colorimetric reaction and found the results within a few seconds. Observation reveals that the limit of detection (LOD) of the adulterants lies in the range between $0.05\%$ (vol./vol.) to $0.2\%$ (vol./vol.) using the colorimetric detection technique. The unknown quantity of the added adulterants is measured using the calibration curves obtained from the experiments results. The repeatability and reproducibility of the process, sensitivity, and the linear range of detection of the calibration curves and the statistical study of the color intensity data are thoroughly analysed herein. In any resource-limited setting, this simple, portable, and user-friendly 3D microfluidic device is expected to be used for testing liquid foods before consumption.

## Introduction

Food adulteration is a serious problem worldwide, and it has received a lot of attention from food safety authorities because it is dangerous to people’s health. Milk is one of the most adulterated food in developing countries, which account for roughly half of total milk production worldwide, including India, Pakistan, China, and Brazil. Milk per capita availability increases year after year, but there is a significant gap between the current growth rate and the required growth rate of milk production. Milk consumption is high because it is a low-cost nutritious food enriched with protein, fat, carbohydrates, vitamins, minerals, etc. Adding adulterants makes the dairy business profitable by bridging the demand-supply gap. The contamination appears to be one of the accessible means of meeting milk consumers’ needs by producing more synthetic milk^1^.

Milk is contaminated with urea^2–4^, melamine^5,6^, detergents^7^, boric acid^8^, formalin^9^, ammonium sulphate, soaps, salt, neutralisers^10^, maltodextrin^11^, starch, sugars^12^, clenbuterol^13^, tetracycline^14^, hydrogen peroxide^15^, caramel, water^16,17^, and many other harmful substances^18^. These chemicals are inexpensive and widely available. The lack of strict enforcement laws and the lack of quick and easy detection techniques are major bottlenecks in keeping this issue under control. Milk quality, typically, is determined by the fat percentage, SNF (Solid Not Fat) value, protein content, and other factors^19^. To improve these parameters, usually, adulterants are added to the milk^1^. Water is added to milk to increase the volume. In contrast, urea and melamine increase the non-protein nitrogen content in milk. Detergents and soaps increase the milk’s whiteness and emulsify the added oil. Hydrogen-peroxide, salt, and formalin are used for the preservation of milk. Sugar and starch are used to increase the density of diluted milk, whereas sodium-hydrogen-carbonate and sodium carbonate are used to neutralize the acidity of the milk. The World Health Organization (WHO) and other food safety authorities’ guidelines specify the safe limit of consumption for these chemicals^20^. The safety limit of urea in milk is 70 mg/100 mL^21^. For hydrogen peroxide and starch, the maximum residue limit (MRL) are $0.05\%$ v/v and $0.15\%$ v/v respectively^22^. For detergent and soap, the safety limit are less than 0.002 mg/kg^23^. According to the Food Safety and Standard Authority of India (FSSAI), milk shall not contain any added amount of salt and carbonates^24^. Consumption of these contaminants above the safe limit can result in harmful diseases such as renal failure, infant death, gastrointestinal complications, diarrhoea, kidney failure, and even cancer in humans^21^.

Different laboratory-based techniques such as lactometer density test, freezing point test, Kjeldahl protein test^25^, Gerber fat test, and others^26^ have been used for a long time to characterise different properties of milk. However, these processes are unable to detect the majority of chemical adulterants. Researchers have developed various detection techniques for various adulterants, such as high-performance liquid chromatography (HPLC), infrared spectroscopy, mass spectrometry, electrochemical signal analysis, fluorescence, admittance, and colorimetric detection with nano-particles^27–33^, among others. On the other hand, these instrument-based laboratory tests are costly and, in many cases, time-consuming. Further, these methods have disadvantages, including weight, availability, energy consumption, and skill requirements. Therefore, the motivation for this work is to design and fabricate a low-cost, accessible device^34^ that can be used in a variety of settings, including household scenarios.

Paper-based microfluidic techniques could be the potential alternative for addressing the disadvantages mentioned above in the context of adulterants detection in milk^35–38^. It is reported that the paper-based microfluidic approaches have used to detect heavy metals^39,40^, antibodies^41^, sulfur dioxide^42^, benzoic acid^43^, D-glucose^44^, formaldehyde^9^, and other compounds in various liquid food samples based on colorimetric detection. Whitesides et al.^45^ created a paper-based device for detecting glucose and protein in urine samples. To give a proper direction to the flow, the authors coated the chromatography papers with hydrophobic agents (SU-8 2010), followed by the exposure to the UV light to make the channels hydrophilic. However, the photo-lithography technique adopted in their study is costly and complicated. Hence, researchers have developed more simpler methods for creating hydrophobic barriers^46^, such as wax printing^47^, toner ink printing using laser printer^48^, plasma treatment of hydrophobic paper^49^, 3D printing^50^, wettability patterning using $TiO_2$^51,52^, using hydrophobic pen^53^, and even using a correction pen^54^. A paper test card is fabricated to detect urea, starch, glucose, and other adulterants in milk using spot test analysis by making hydrophilic detection spots^12,55^. Using wax printing technique^56–58^, PDMS coating^59,60^, etc., hydrophobic barriers are made to store the chemical reagents on the hydrophilic circular zone. After a few minutes, the colorimetric reactions are captured on cell phones, and image analysis was used to determine the amount of added adulterants^39,43,44,61,62^. These methods have drawbacks such as low resolution, high cost, unavailability of the wax printer, hydrophobic barrier strength, lack of simultaneous detection, and so on. On the other hand, the ink-jet printing method^63^ is simple, quick, and inexpensive, but the barrier strength is insufficient to restrict the flow of reagents within the detection zone for different liquid samples. These various methods of fabricating hydrophobic barriers demonstrate the importance of research to develop a simple fabrication technique. Despite several advantages mentioned earlier, paper-based devices suffer from several issues. The paper’s porous structure often decreases the liquid transport to the desired location due to spreading the liquid in all directions. Other disadvantages related to paper-based microfluidic devices are loss of sample due to evaporation, sample pretreatment step, low sensitivity, high LOD, less shelf life of stored reagents, and most importantly, lack of commercialisation^64^.

Here, we have created a novel design for a 3D, portable, low-cost paper-based microfluidic device that can detect multiple adulterants simultaneously in a liquid sample using a small volume of liquid sample (1–2 mL). The liquid flow is caused in the porous paper substrates due to the inherent capillary action. As no (super)hydrophobic coatings are used, the device is durable and can be used for adulterant detection in many liquid foods. The patented (App. no 345721-001) design of the 3D device ensures that the liquid flow rate remains the same as that of the pure paper substrate. A few cuts are made in the middle supporting layer to reduce the resistance of the liquid flow on the paper substrate, and the velocity of the liquid flow remains the same as in the pure paper case. The colorimetric detection technique identifies the adulterants in the detection zones, and quantitative analysis has been performed using a color intensity test. For the first time, we demonstrated that the device could detect urea, detergents, soap, starch, hydrogen peroxide, sodium-hydrogen-carbonate, and salt simultaneously in milk samples. The patented (App. no 202141024502) simultaneous adulterants detection technique for milk samples is better than the conventional single strip-based detection where multiple experiments are required to identify one adulterant. The amount of added adulterants in milk is quantified using the color intensity test, with detection limits ranging from $0.05\%$ (vol/vol) to $0.2\%$ (vol/vol) for different adulterants using the device. A quick and straightforward fabrication technique makes the device suitable to use in resource-limited settings. The device’s design is scalable, which allows the number of detection spots to be easily altered. Furthermore, using the device’s current design, we discovered the detection limit close to the existing instrument-based detection techniques. Therefore, this device will fulfill the ASSURED (Affordable, Sensitive, Specific, User friendly, Rapid and Robust, Equipment free, Delivered to those who need them) criteria by addressing both the technical (ASSR) as well as user acceptance (UED) aspects together.

## Results and discussion

### Adulterants identification

**Figure 1:**
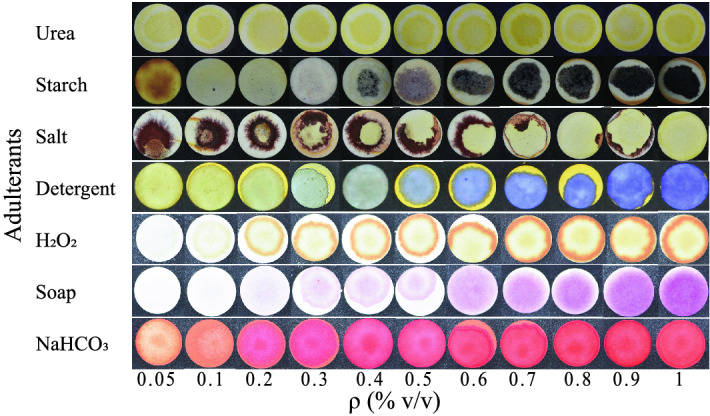
The change in color after the colorimetric reaction for different concentration of adulterants has been shown for all the adulterants.

Different adulterants are detected using a paper-based microfluidic system via colorimetric techniques. Table S1 shows the color variations of the detection zones in the presence of different adulterants. Figure 1 shows the color shift as the concentration of added adulterants is increased. The illustration shows that when the concentration of adulterants rises, the intensity of the particular color also increases. The appearance of non-uniform color patterns in the detecting zone is due to chemical reagents’ deposition after the solvent evaporation. Controlling the evaporation flux and internal Marangoni/buoyancy flow to create a uniform color pattern in the detecting zone is beyond the scope of this research. However, the color intensity variation along the radius of the circular spots is shown in Fig. S1. It is easily visible that there is not much variation for the lowest concentration, while for the higher concentration, the color variation is more. We found that the color intensity decreases from the center to the radial direction for some adulterants. However, the intensity increases from the center to the radial direction for hydrogen peroxide and urea. Color intensity tests are used to quantify the amount of additional adulterants. Small circular hydrophilic spots on paper are produced to keep the chemicals in the spot test platform. The pure milk is then mixed with varying quantities of adulterants ($\rho$) and poured into the circular spot using a syringe. All of the spots have 10 $\upmu$L of reagents and 20 $\upmu$L of samples. Urea, detergents, starch, salt, $H_2O_2$, $NaHCO_3$, and soaps are all tested at room temperature. The detecting spots changed their color as soon as the sample came into contact with the reagents. There is no time delay to change the color in the detection spots in the presence of the adulterants. To check the repeatability of the color intensity for the same concentration, we performed the Anova test on the intensity values. In Table S2, we have shown a summary of the one-way Anova test where the null hypothesis of considering all the means of color intensity values are the same is accepted.

**Figure 2:**
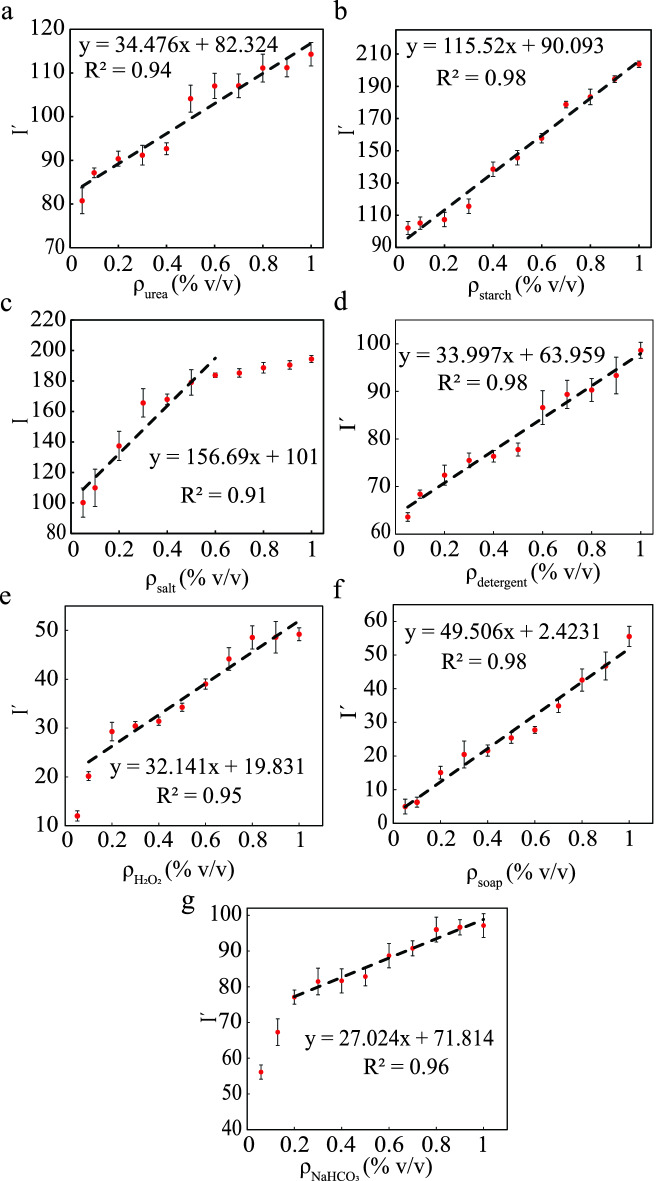
Color intensity curves are shown for eight different adulterants with varying concentration from $0.05\%$ to $1\%$ (v/v) added in milk for (**a**) urea, (**b**) starch, (**c**) salt, (**d**) detergent, (**e**) hydrogen peroxide, (**f**) soap, and (**g**) sodium-hydrogen-carbonate. From the figure it is clear that with increasing concentration the color intensity is also increasing.

The average color intensities (I) of the detection zones are determined using image processing techniques. Using ImageJ application, the red (R), green (G) and blue (B) intensity values are obtained^48^. The normalised gray intensity values are calculated for all the adulterants except salt using Eq. (1) and for salt using Eq. (2).

1 $$\begin{aligned}I '= 255-0.299R-0.587G-0.114B \end{aligned}$$

2 $$\begin{aligned}I = 0.299R-0.587G-0.114B \end{aligned}$$

The color intensity curves for all of the different adulterants with varying concentrations ($\rho$) are given in Fig. 2. In most situations (except salt where the color change happens from low (brown) to high (white) intensity), we employed reverse intensity values ($I '=255-I$) to depict the specific color in ascending order as it transitioned from a high-intensity to a low-intensity color. We did a quantitative investigation of an unknown amount of added adulterants in milk using these color intensity curves. Table S3 contains the calibration curves discovered via curve fitting $(R^2>0.95)$ from the color intensity graphs. However, to perform the quantitative analysis we have used the classical linear regression fit curves which is shown in Fig. 2. With five repeated experiments at every different concentrations the color intensity curves are formed and the error bars are represented as the standard deviation of the multiple experiments. The more details about the color intensity analysis is described in the error analysis subsection.

Linearity in the predicted working range should be validated for any calibration curves. For most of the cases in our study, linear range is the same as the dynamic range, so this process gives more accurate and precise data^65^. In most of the cases, the curves are linear as the regression values are coming more than 0.9 in the range of $0.05\% (v/v)$ to $1\% (v/v)$. The detail investigation of the linear range study is shown in Fig. 2. Here, we have mentioned the visible limit of detection (LOD) qualitatively from the colorimetric reaction. The intensity of the color changes is identified using ImageJ, which is shown in Fig. 2. The minimum noticeable change in the color intensity of each case is considered as the LOD of the device^66^. We discovered that the LOD of these adulterants using the colorimetric detection methodology is nearly identical to that of the conventional methods. The sensitivity of the linear responses and the comparison of the LODs of existing processes with the current work are summarised in Table 1. However, there is a need for further improvement in the LOD to use the device for field-based assay, as the maximum residue limit (MRL) is less than the LOD of the paper-based device. This device is not sufficiently sensitive to detect the very low quantity of adulterants in milk samples. To further improve the device’s sensitivity, it can be integrated with electrochemical and colorimetric detection, which is the future scope of this study.

**Table 1:** Comparison of the limit of detection of the colorimetric technique for the adulterants in the present work and the existing different techniques.

Adulterants	Linear range ($\% v/v$)	Sensitivity	LOD (v/v) (present work)	LOD (v/v) (published work)
Urea	0.05–1	34.476	$0.05 \%$	$0.05 \%$^67^, $0.025 \%$^68^, $0.3 \%$^69^
Starch	0.05–1	115.52	$0.1 \%$	$0.17 \%$^59^
Salt	0.05–0.6	156.69	$0.1 \%$	$0.29 \%$^59^
Detergent	0.05–1	33.997	$0.2 \%$	$0.2 \%$^59^
$H_2O_2$	0.1–1	32.141	$0.1 \%$	−
Soap	0.05–1	49.506	$0.2 \%$	−
$NaHCO_3$	0.2–1	27.024	$0.2 \%$	$0.1\%$^70^

**Figure 3:**
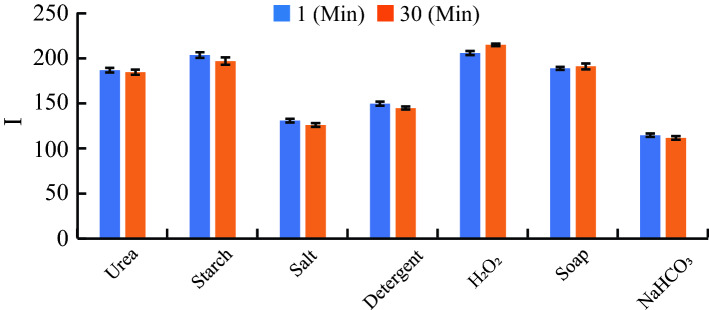
The color intensity values of the detection zones are shown here for 1 min and 30 min.

The color intensity is also tested for stability over time. The color intensity differences for all detection zones are shown in Fig. 3 at the start and end of 30 min. In the 30 min time interval, we observed that the maximum difference between color intensities at start and end is found $4.8\%$ for hydrogen peroxide adulterant. It is clear from these results that users can take images after some time for performing the test and hence the absence of instant imaging has no effect on the outcomes.

### Detection of adulterants simultaneously using microfluidic paper-based chip platform

**Figure 4:**
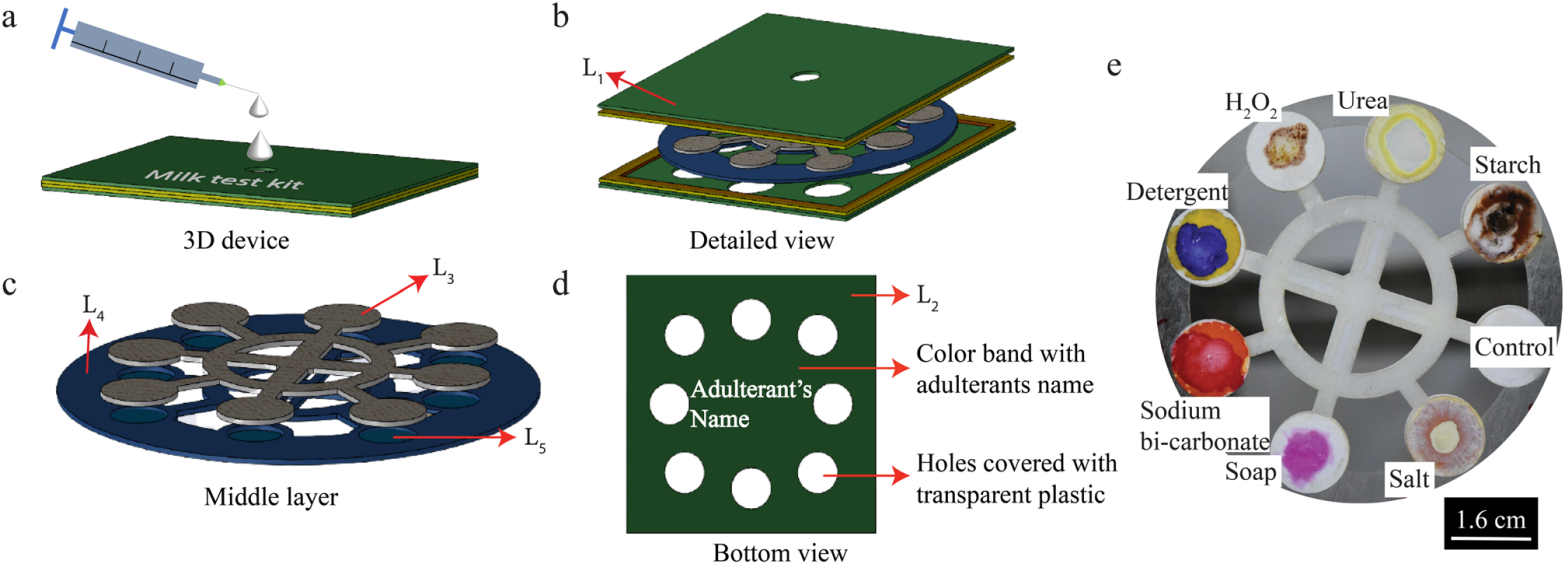
The schematic representation of (**a**) the compact device. Sample is added to the device through the hole in the top cover, for testing. (**b**) Device’s detailed view. Three layers have been shown here as the top cover $(L_1)$, 3*D* paper-based microfluidic device, and the bottom cover $(L_2)$. (**c**) The double layers 3*D* paper-based microfluidic device is shown here. This is a sandwich structure where solid support is sandwiched between two layers of filter paper. $L_3$ represents the transportation zone, $L_4$ represents the solid plastic layer, and $L_5$ represents the detection zone. (**d**) Design on the backside of the bottom cover. Adulterants name and a color band are given for qualitative and quantitative identification. (**e**) Image of simultaneous detection of the seven adulterants using the 3D paper-based microfluidic device is shown (only the middle layer).

**Figure 5:**
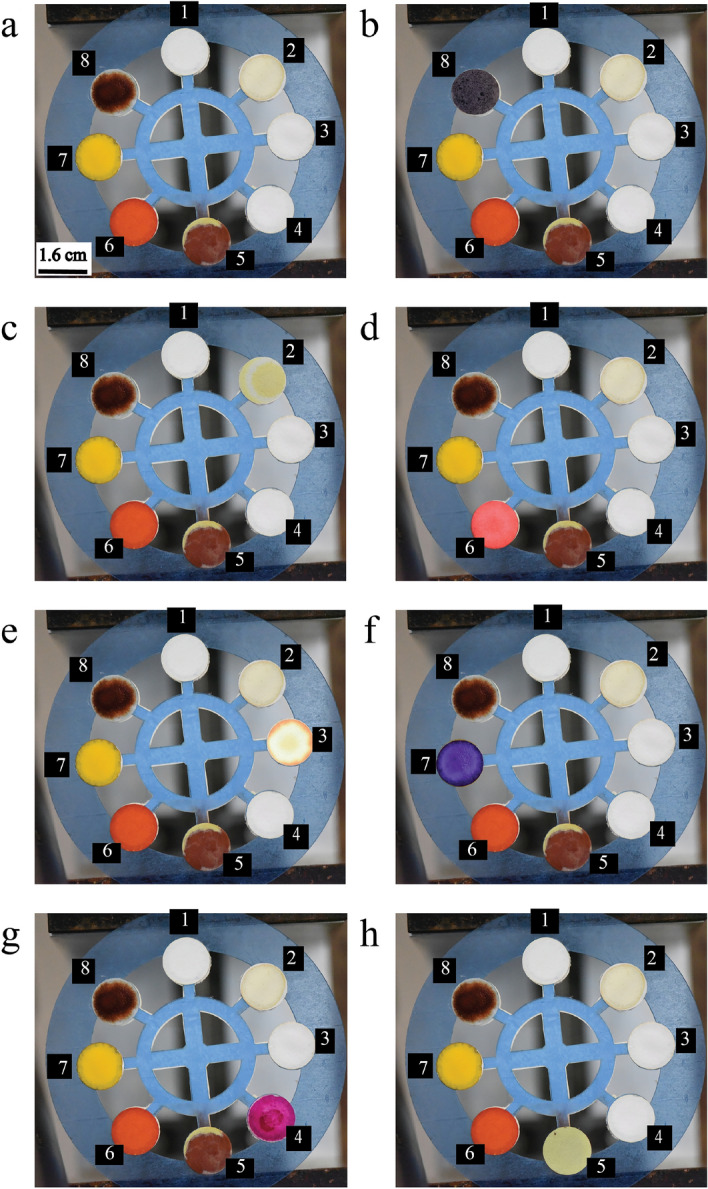
Experimental results of specificity test. (**a**) Only the reagents are showing in the different detection zones where the number represents the detection zones for urea (2), $H_2O_2$ (3), soap (4), salt (5), $NaHCO_3$ (6), detergent (7), starch (8) and control (1). Specificity of the reagents have been shown for (**b**) starch, (**c**) urea, (**d**) $NaHCO_3$, (**e**) $H_2O_2$, (**f**) detergent, (**g**) soap, and (**h**) salt.

**Figure 6:**
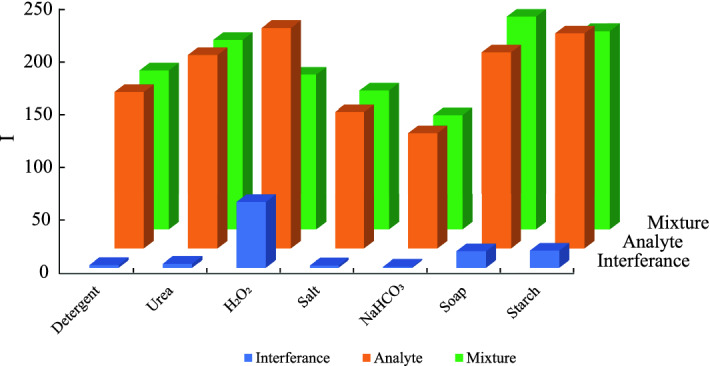
The difference in color intensity of the adulterant mixture and the single adulterant is shown here. The maximum interference is found at about $30\%$ for hydrogen peroxide but for all other cases it is less than $10\%$.

A 3D design is made and fabricated the device to simultaneously perform the multiple adulteration test. The schematic of the 3D device is shown in Fig. 4. Figure 4a depicts the compact device’s schematic, whereas, Fig. 4b,c depict the device’s detailed view and the sandwich structure middle layer, respectively. Figure 4d shows the design of the bottom cover. In Fig. 4e, the experimental image is shown where the simultaneous detection of seven adulterants is performed in the middle layer. Studying the specificity of the reagents and interference’s effect on the color intensity of different adulterants is critical for simultaneous detection of adulterants in the 3*D* device. We added one specific adulterant to each reaction zone for specificity analysis. In addition to the detection zones, we have created a control zone where no reagents are stored. We discovered a nearly negligible change in color intensity in the control zone for pure and adulterated samples. As a result, we assumed that the color intensity value of milk samples in the control zone is 255 because it appears pure white. It is found that there is a color change in a specific location for the specific adulterant only. Figure 5 shows a detailed specificity study of all adulterants. All experiments are carried out at a temperature of $25 \pm 2$ $^\circ$C. We prepare the adulterated samples in distilled water using each of the adulterants separately and then add them to all of the detection spots on the device. Based on these results, we can conclude that the reagents are specific to a single adulterant or a group of adulterants, as they do not change color in the presence of other chemicals. We also used pure milk to see if there was any color change in the detection spots. However, the reagents do not affect the color of pure milk. These reagents do not react with any pure milk ingredients and react only when the adulterants are present, followed by the color change. Thus, the color interference test is carried out by considering only those few adulterants. We determined from the interference test that there is little change in color intensity during simultaneous detection due to the interference effect of multiple adulterants. For the interference test, two different milk samples of the same volume are prepared with the same amount of added adulterants. In one sample, all of the adulterants are mixed, while only one adulterant is added in another. The colorimetric detection of the mixture and single adulterant is then performed to determine the change in color intensity. Figure S2 reveals the colorimetric detection of adulterants for both a mixture of adulterants and a single adulterant. Figure 6 shows the difference in color intensity values of the single adulterant and the mixture of the adulterants. Color interference is defined as the difference in color intensity between the intensity of the single analyte and the mixture of adulterants. The comparison of the color intensities shows that the interference effect of adulterants on color intensity is significantly less. As a result of its reasonable specificity and minor change in the color intensity due to interference, simultaneous detection of adulterants on the paper-based microfluidic platform is entirely possible.

**Table 2:** Quantitative detection of added adulterants in milk samples.

Adulterants	Added amount (mg)	Found amount (mg)	Recovery (%)	RSD ($\%$)
Urea	55	58.4	106.20	2.18
Starch	110	96.88	88.08	3.51
Salt	80	79.12	98.9	1.51
Detergent	45	44.99	99.98	0.80
$H_2O_2$	85	90	105.90	0.72
Soap	65	55.62	85.57	0.50
$NaHCO_3$	95	86.56	91.10	1.93

Experiments are carried out using the 3D $\mu$PAD for the simultaneous detection of multiple adulterants in one step. Figure 4e shows the colorimetric detection of multiple adulterants in a single device. For a submicron volume of sample dispensing requires a dedicated setup, which may increase the device’s cost. Also, it will increase the operational cost and may be helpful for knowledgeable users. On the other hand, colorimetric detection requires a specific volume of reagents. The expected distinct color change will not be appreciable in the case of a low sample or reagent volume. Therefore, after several trials, we have used 10 $\upmu$L reagents in all the detection spots in this study. For the simultaneous detection of adulterants in the milk sample, an average volume of 1.5 mL milk is used in different experiments. However, future studies are required to optimize the sample/reagents volume, and we must implement the corresponding design modifications to easily measure and dispense exact quantity of samples. It is found that the color change typically occurs as soon as the sample enters the detection zone. Using the previously mentioned calibration curves shown in Fig. 2, we determined the added amount of adulterants in milk samples for quantitative analysis. Before dispensing the milk on the device, different amounts of adulterants are added to the milk sample. After the colorimetric reaction images are taken using a camera. Color intensities are extracted from these images and entered into calibration equations to calculate the added amount. In the calibration curves mentioned in Fig. 2, X represents the percentages (v/v) of adulterants detected in the milk, and Y represents the color intensity values of the detection spots following the colorimetric reactions. Table 2 shows a comparison of the actual amount of added adulterants, the results of the calibration curves, and the device’s accuracy. We have obtained the recovery rate (RT) of the paper device using the relation^14,40,71,72^, $RT = \frac{\text {Amount recover}}{\text {Amount added}}\times 100\%$. Using the device, the recovery percentages of the added adulterants are found to be in the range of 85–107% with a good reproducibility of 0.50–3.51% (RSD = Relative standard deviation) after multiple experiments. Using the color intensity detection method, we achieved detection accuracy of more than $80\%$. The supplementary document shows the real image of a used compact device in Fig. S3. We have also added a video of simultaneous detection of the adulterants in milk samples using the 3D paper-based microfluidic device in the supplementary video S1.

Finally, a cost estimation is carried out for fabricating the 3D paper-based microfluidic device. The prices of the chemicals, papers, and solvents for a specific amount are known. Hence, we divided the total cost of the specific amount to determine the cost of the used amount. The price of a box of 100 Whatman filter paper (grade 4) is Rs. 1300 ($16.39\$$), and we used one paper to make one device. As a result, it will cost Rs. 13 ($0.16\$$). Similarly, some amount of the reagents are used to make 10 mL solution with different solvents, and only 10 $\upmu$L of that solution is used to make the device. The pricing for creating a device is listed in Table S4. The solvent price is included in the reagent price. According to the cost analysis, the approximate price for one device is Rs. 17.70 ($0.23\$$). A detailed comparison of cost, LOD, testing time with the traditional methods are described in the supplementary document in Sect. S8.

## Conclusion

In the present work, a paper-based (Whatman filter paper grade 4) device is fabricated and used for detecting multiple (seven) adulterants in milk samples simultaneously based on the colorimetric technique. Observation reveals that only 1–2 mL of sample volume is required for each test, and the testing time is less than 30 s. Qualitative and quantitative analyses are carried out, with a recovery range of 85–107% and RSD range of 0.50–3.51% using the developed calibration curves. The adulterants’ linear range, sensitivity, and LOD are close to the existing methods. The cost of testing for seven adulterants is approximately $0.23\$$ only. The method is reliable for detecting adulterants in milk on the spot. Further, the lightweight, low-cost, simple-to-use, and environmentally friendly method makes this device suitable for inspecting many liquid foods. It is inferred from the investigation that the reagent only reacts with the specific adulterant in this method and not with any milk ingredients. Hence, this analytical tool can help to monitor liquid food safety and thereby increases the traceability of tainted milk in remote areas of developing countries.

It should be noted that by appropriately modifying the chemical reagents, the current device can be used not only for milk but also for water, protein shakes, fruit juices, etc. Further, the future aim is to use a mobile application to perform quantitative analysis to determine the adulterants’ concentration. Emphasis will also be given to addressing the possible limitation of the developed device, e.g., reagent evaporation effect, a common platform for finding the color intensity in different brightnesses, detecting any unknown adulterants using artificial intelligence, and so on.

## Methods

### Materials and apparatus

Chemicals such as para-dimethylaminobenzaldehyd (p-DMAB), phenolphthalein, bromocresol purple, iodine solution, potassium dichromate, silver nitrate, potassium iodide, rosolic acid, hydrochloric acid, and ethanol are provided by Sigma Aldrich, as well as different adulterants such as urea, hydrogen peroxide, sodium-hydrogen-carbonate, and Whatman filter paper grade 1 and 4 (rectangular and circular shape). Soap, detergents, starch powder, salts, different milk samples (commercially available toned milk samples containing $3\%$ fat), photographic paper, PVC sheets, and other items are procured from local shops. All the chemicals are used without any further purification.

### Reagent and sample preparation

**Figure 7:**
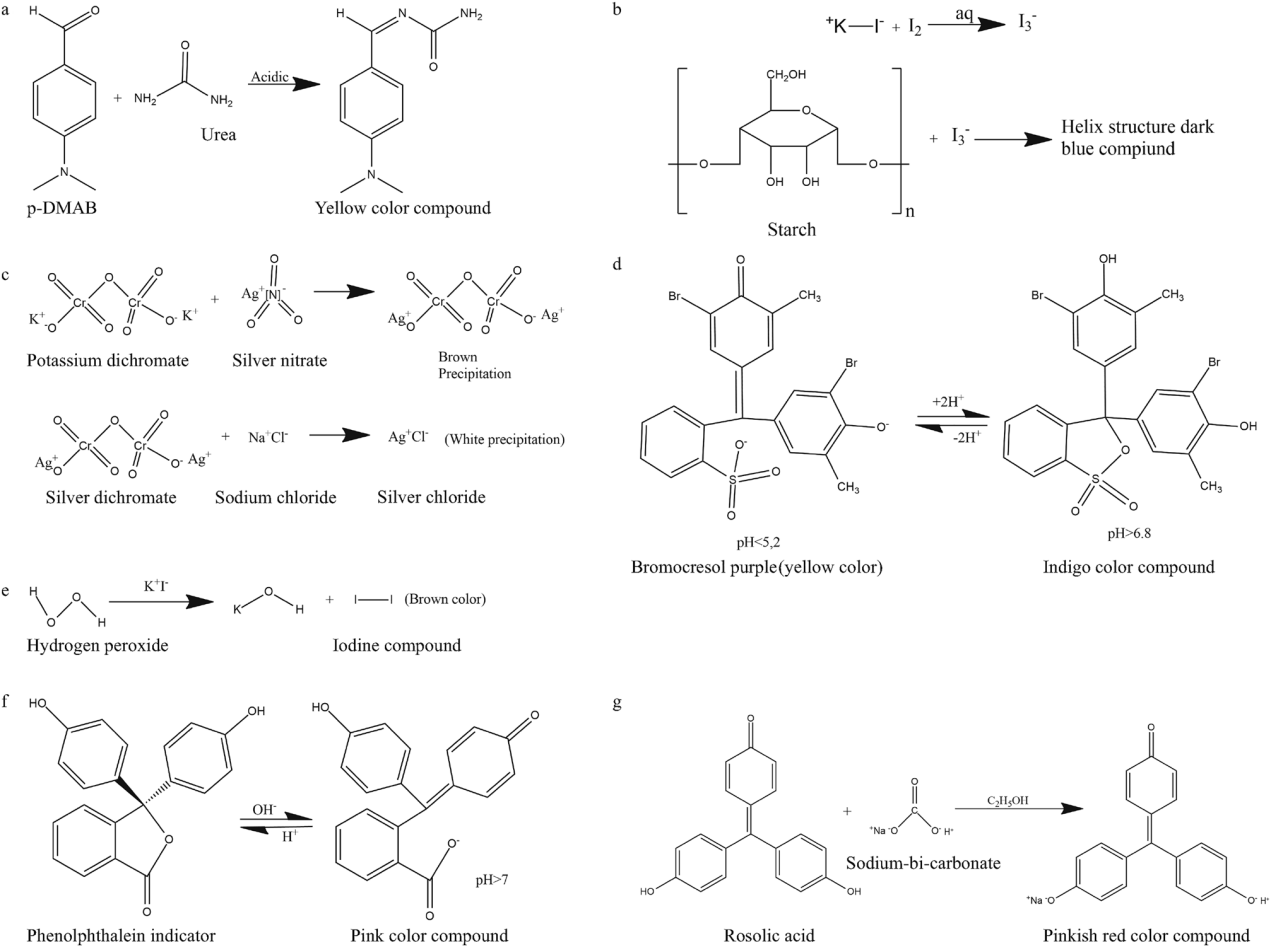
Schematic representation of the colorimetric reactions of (**a**) urea^73^, (**b**) Starch^74^, (**c**) salt^75^, (**d**) detergent^76^, (**e**) $H_2O_2$^77^, (**f**) Soap^78^, and (**g**) $NaHCO_3$^79^.

All the reagents are dissolved either in distilled water or in ethanol, depending upon their solubility. Using colorimetric detection techniques, all the adulterants are detected in different liquid samples. For detecting urea, a $1.6\%$ (w/v) p-DMAB solution is prepared by dissolving it in a $10\%$ (v/v) concentrated hydrochloric acid solution in ethanol. To detect soap and detergents, $1\%$ (w/v) phenolphthalein reagent solution dissolved in ethanol and $0.5\%$ (w/v) bromocresol purple solution dissolved in water are used, respectively. $1\%$ (w/v) iodine added to a $10\%$ (w/v) potassium iodide solution dissolved in water is made to detect starch. For detecting salt, we have used precipitation produced by mixing one drop of 0.1 N potassium dichromate solution and 1 N silver nitrate solution dissolved in water. A saturated potassium iodide solution dissolved in water-ethanol (3 : 1 v/v) solution is used to detect $H_2O_2$. Neutralisers like sodium-hydrogen-carbonate were detected using $1\%$ (w/v) rosolic acid solution dissolved in ethanol. We have added a drop of contaminated milk to the detecting zones after adulterating it with different chemicals to check the colorimetric reactions. Urea interacts with p-DMAB in an acidic media to produce a yellow chemical (Fig. 7a). Figure 7 shows a schematic illustration of chemical reactions. We ran this test with various urea concentrations to see whether there are any differences in color intensity, and we found with an increase in urea concentration results in an increase in yellow color intensity. Different adulterants are also introduced to milk and colorimetric detection is carried out. The iodine solution combines with starch to produce a blue compound (Fig. 7b). In the presence of detergent, bromocresol indicator gives an indigo hue in a less acidic media (Fig. 7d), and salt reacts with silver dichromate precipitation to give a white silver chloride precipitation (Fig. 7c). A brown hue iodine compound is formed when hydrogen peroxide combines with saturated potassium iodide solution (Fig. 7e). In the presence of soap, phenolphthalein takes on a pink color in the less acidic medium, and sodium-hydrogen-carbonate combines with the alcoholic rosolic acid solution to generate a pinkish-red molecule (Fig. 7g). All the measurements are performed using a weighing balance (Pioneer, Ohaus) of 1 mg accuracy, a measuring jar, and a pipette. For testing liquid food samples, we have used distilled water and toned milk. For spot test analysis, different concentrations of an adulterant is added to 10 mL of liquid samples. For simultaneous detection of multiple adulterants, we added different adulterants to a 10 mL milk sample. Due to the addition of more solid compounds in milk, its density gets increased and hence a much higher value in the lactometer reading is found in comparison to the pure milk. Therefore, we added distilled water to the sample to dilute it so that the lactometer reading remains comparable to that of the pure milk. The details of the sample preparation is described in the supplementary document in Fig. S4.

### Design and fabrication of the spot test platform

We used spot test analysis to perform the color intensity test and determine the limit of detection (LOD) of the measurements. The small circular patterns of 16 mm diameter are created using the drawing software AutoCAD 2021 and printed on a commercial HP laser printer (Laser Jet Pro MFP M227fdn). The diameter of the circular zone is calculated by equating the liquid’s dispersed volume (40 $\upmu$L) with the height and area of the circular zone. To make the printed paper hydrophobic, the pores on both sides of the paper should be blocked. So the paper is heated on a hot plate (Spinot) for 20 min at $170 \pm 1$ $^\circ$C to block the pores on the other side^48^. The ink melted and spread through the pores to the other side of the paper as a result of the heating. To stabilise the coating, the paper sheet is left at room temperature for 5 min. We measured the contact angle of the hydrophobic barrier with a Goniometer (Holmarc), which is $120\pm 2^\circ$.

### Design and fabrication of the 3D $\mu$PAD

We have used an universal $CO_2$ laser system (10.6 $\upmu$m $CO_2$ Laser, 60 W power) (used $3\%$ of maximum speed, $3\%$ of maximum power for paper, and $11\%$ of maximum power and $2\%$ of maximum speed for plastic) to cut the papers in the desired shapes. The 3D paper-based microfluidic device is made up of a top and bottom cover ($100 \times 100$ mm$^2$) and a sandwich structure middle layer. One small hole of 10 mm diameter in the top cover is provided to add the sample. The bottom cover has multiple holes (16 mm diameter) for inspecting the findings after trials. The sandwich structure paper device has one plastic layer sandwiched between the two layers of paper to improve the strength of the device. On the top paper layer, there is a sample inlet point (10 mm in diameter), primary tracks ($10 \times 6$ mm$^2$), ring track (5 mm in width), secondary tracks ($5 \times 4$ mm$^2$), and a vertical transportation zone (16 mm in diameter). The plastic layer supports the paper and has several cuts ($10 \times 2$ mm$^2$) and holes (12 mm diameter) to improve velocity and transmit the sample to the second layer of the paper (Fig. S5). The detection zone (16 mm diameter) on the bottom paper layer is where the detecting agents will be loaded. Double-sided tape and glue are used to link the transportation and detecting zones to the plastic layer, respectively. This 3D design works well for transporting denser liquids at a consistent speed. The number of tests can be raised by altering the ring track’s size. During the tests, substances are poured into the small opening on the top cover (1–2 mL) and moved to the detecting zone via the paper tracks due to capillary action. A transparent cover is provided on the outside of the device to reduce the rate of reagent evaporation. The paper is treated with reagents and let to dry. Both paper layers are adhered to both sides of the support after drying, and the covers are adhered with double-sided tape. On one side, we keep the reagents, and on the other, we keep the samples. The sample comes into contact with another layer through the support holes due to the vertical mobility of the liquid. The images of the detection zones are taken by a DSLR camera (Nikon D750) with a 105 mm focal length lens, by maintaining a constant lens aperture of f/4.5, ISO 1000, and a resolution of $2624 \times 3936$ pixels every time. Because of its high porosity (avg. pore size of 25 $\upmu$m) and thickness (210 $\upmu$m), Whatman filter paper grade 4 is used in this design, which aids liquid flow and allows for the storage of more reagents. We compared the qualities of grade 4 paper with those of grade 1 filter paper (primarily used in research, 11 $\upmu$m pore size, 180 $\upmu$m thickness) by running multiple liquid flow experiments through both types of paper. The results demonstrate that the flow is faster on Whatman grade 4 filter paper than on grade 1 (Fig. S6).

Using this design, we have overcome certain disadvantages of the previous studies. Simultaneous detection of multiple adulterants is performed using the proposed 3D device. We have not used any hydrophobic material to make the hydrophilic path and different circular spots in the current device design. Therefore, there is no problem of reagents leakage from the device due to the weak hydrophobic barrier strength. Our proposed method is facile and scalable by eliminating the hydrophobic coating step. Using two layers of paper for transportation and detection, we have solved the cross-contamination of the reagents. Another advantage of making a two-paper-layer 3D structure is that the liquid flow is not interrupted due to the blockage of the paper pores. If one layer of paper is used to perform the experiments, then back-flow of the reagents through the paper channels will happen and eventually block the pores. Therefore, we made a 3D design where the flow of liquid samples and storing of reagents were performed in two separate layers, and the sample reached the detection zones from the top side.

### Design selection

In the 3D design (Fig. 4c), the top paper layer is attached to a plastic support at the bottom whose thickness is similar to the paper thickness. So the liquid flow must be interrupted due to the resistance force at the bottom side of the paper. We have compared the liquid flow of three different cases and considered the optimal case for strength and velocity. Paper strips ($30 \times 6$ mm$^2$) are taken and multiple experiments are performed using a paper holding setup. As the liquid flows through the whole horizontal paper section within 30 s, in the case of only paper, the conventional Lucas-Washburn analytical model (without gravity) is applicable^16,80^. In this model, the force balance is considered between the capillary force and the viscous resistance force, where the capillary height (L) is related to time (t) as, $L \propto t^{0.5}$. We named this only paper case Type 1 and found that it is satisfying the conventional relation.

**Figure 8:**
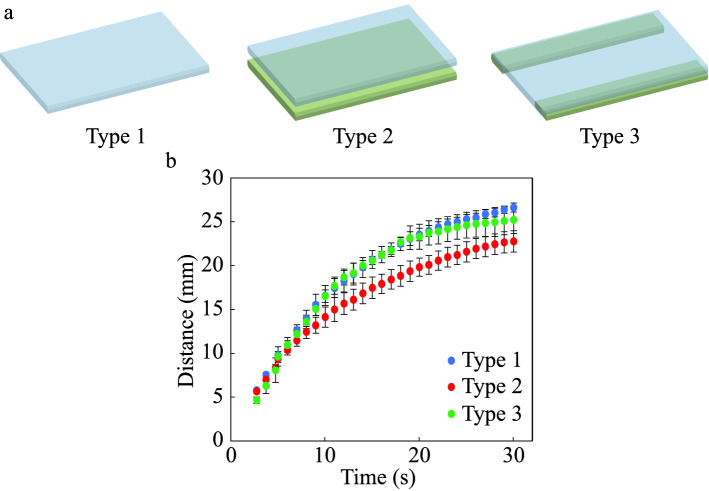
The schematical representation of the supporting layer is shown here, (**a**) for the three cases, Type 1, Type 2, Type 3. (**b**) Comparison of the experiments of distance travel by the liquid in all the three different cases.

Type 1 is better than the other possible cases, as there is no resistance force acting at the backside of the paper. Following that, we looked at two more cases where paper with plastic support on the backside is named Type 2 and paper with plastic support with a channel cut in the backside is named Type 3. The schematic representation of all the three cases are illustrated in Fig. 8a. In each of the three examples, many liquid flow tests are carried out. The outcomes of the experiments are compared in Fig. 8b. We discovered that the flow velocity is about identical in Type 1 and Type 3, but it is slightly lower in Type 2 due to high resistance. As a result, we chose Type 3 as the best scenario because it provides strength because of the plastic layer and has a similar flow velocity to Type 1 due to the decreased resistance force caused by the cuts.

### Error analysis

**Table 3:** Specification of the measurements.

Measured parameters	Uncertainty
Volume	$\pm 2\,\,\upmu$L
Weight	$\pm 0.001$ gm
Length	$\pm 0.05$ mm
Color intensity	$11\%$

**Figure 9:**
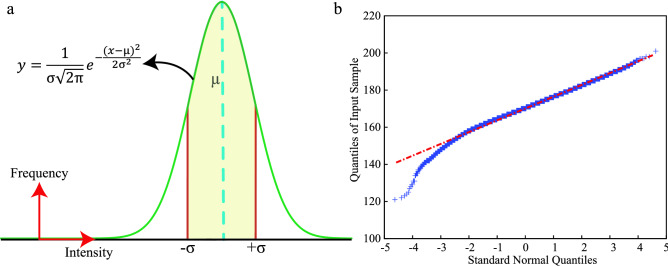
The graphical schematic of the statistical analysis is shown here. (**a**) The $\pm \sigma$ range under the Gaussian distribution curve is shown here. (**b**) Q–Q plot of a particular set of experiments is shown here to verify the normal distribution trend of the color intensity values.

A detailed error analysis is performed and listed in Table 3. Here we showed the instrumentation and systematic error for measuring the volume, weight, length, and color intensity. Volume is measured by measuring jar and pipette, which have instrumentation uncertainty. Weight is measured by weighing balance which also has instrumentation uncertainty. We considered instrumentation uncertainty in measuring the length using a laser cutter. For repeated experiments, systematic uncertainty is considered for measuring the color intensity. We selected the point with the maximum error bar from all the data points and found the percentage uncertainty for the same.

Further, the color intensities of the detection zones for all the adulterants are analysed statistically. All the color intensity data show the normal distribution trend. To get the average value of color intensities from the repeated experiments, we have considered the data under the $67\%$ area of the Gaussian distribution curve. The schematic representation of the Gaussian curve is shown in Fig. 9a. We also performed the Quantile–Quantile (Q–Q) plot analysis to check the Gaussian trend. A representation of the Q–Q plot for sodium-hydrogen-carbonate is shown in Fig. 9b, where the tail is not matching with the straight line. We found that the color intensity Gaussian distribution is right-skewed and left-skewed, but the main body matches the Gaussian distribution. The scattered data plot of a few adulterants for all the different concentrations is shown in Fig. S7. We have also performed the Bayesian regression analysis with all the data points. A details description of the Bayesian regression analysis for a few adulterants is described in the supplementary documents S1. The Bayesian regression table for the adulterants is shown in Table S6.

## Supplementary Information

Supplementary Information 1.

Supplementary Information 2.

## Data Availability

All data generated or analysed during this study are included in this published article [and its supplementary information files]. The datasets used and/or analysed during the current study is also available from the corresponding author on reasonable request.
