# Supplementary material for: 3D Paper-based milk adulteration detection device
Source: Sci Rep. 2022 Aug 11;12:13657. doi: 10.1038/s41598-022-17851-3 (PMC9372070; doi:10.1038/s41598-022-17851-3)
Supplement: Supplementary file 1 — Supplementary Information 1. [file 41598_2022_17851_MOESM1_ESM.pdf]

Supplementary Information

3D Paper-based milk adulteration detection device

Subhashis Patari[a], Priyankan Datta[a], and Pallab Sinha Mahapatra[a,1]

[a]Micro Nano Bio-Fluids group, Department of Mechanical Engineering, IIT Madras, Chennai 600036, India

[1]Address correspondence to: Pallab Sinha Mahapatra

Department of Mechanical Engineering

Indian Institute of Technology Madras

Chennai, Tamil Nadu 600036, India

E-mail: pallab@iitm.ac.in

S1. Color change

In the presence of different adulterants different color is appeared in the detection zones. The color changes before and after the colorimetric reaction is listed here in Table S1.

Table S1: Color change in the detection zone due to the presence of adulterants.

Adulterants	Color if positive	Color if negative
**Urea**	Yellow	White
**Soap**	Pink	White
**Detergents**	Indigo	Yellow
**Starch**	Blue	Brown
**H_2O_2**	Brown	White
**Sodium-hydrogen-carbonate ($NaHCO_3$)**	Pinkish red	Orange
**Salt**	White or yellow	Brown

S2. Color variation

The color variation of the detection zone is shown in Fig. S1. With a color bar scale the color change is shown for all the different adulterants. The color variation for the lowest concentration of adulterants and maximum concentration of adulterants are compared here. We found that the color intensity is varying along the radius of the circular detection spots. For some adulterants from the center to outer perimeter the color intensity is decreasing and for some adulterants it is increasing. From this study we can identify the effective area in the detection zone to check the presence of adulterants.

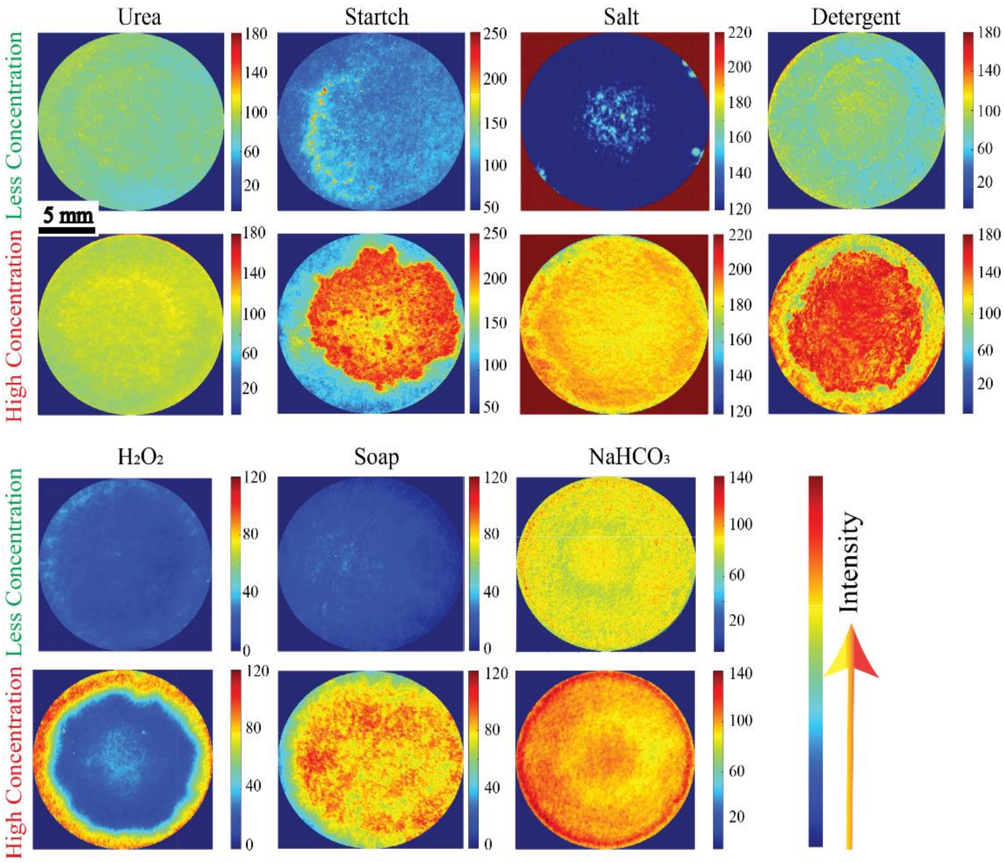

Figure S1: Color bar representation of the color intensity variation along the radius of the circular spot is shown here.

S3. Repeatability test

In this study we have performed multiple experiments for every different concentration of adulterants to find out the average intensity value. Due to the multiple experiments repeatability of the results is important. Here we have performed the one-way Anova test to check the repeatability of the experimental results. We have considered a null hypothesis that the groups mean are equal and an alternative hypothesis that the groups mean are not equal. In the Table S2 we have given the details of a set of experiments and performed the one-way Anova test to check the repeatability.

Table S2: Anova Single Factor

SUMMARY

Groups	Count	Sum	Average	Variance
1	10	1725	172.5	9.166667
2	10	1725	172.5	9.166667
3	10	1755	175.5	9.166667

ANOVA

Source of Variation	SS	df	MS	F	P-value	F crit
Between Groups	60	2	30	3.272727	0.053377	3.354131
Within Groups	247.5	27	9.166667			
Total	307.5	29				

The result is "Null hypothesis accepted".

Our null hypothesis is all the mean in the different groups are same. Here in the 1st table summary of the input data are given. In the 2nd table the details of the one-way Anova test are listed. The variation of the data between groups and within the groups is listed here. SS represents the sum of squares which indicates the variability between and within the groups. df is the degree of freedom which is defined for between groups as: (number of groups – 1) and for within groups as: (number of counts – number of groups). MS is the mean square which is define as the ratio of SS and df. F statistic is represented as the ratio of MS (between groups) and MS (within groups). For accepting the null hypothesis F statistic must be less than F critical which is satisfying in this case. Also, the P-value is more than the significance level. So, our null hypothesis of considering all the mean in different groups are same is accepted with a confidence interval of 95%.

S4. Calibration curves

Here the higher order calibration curves are listed for different adulterants to find out the unknown amount of the added adulterants.

Table S3: The details equation of the calibration curves for all the adulterants is given here with the regression value.

Adulterants	Calibration curve	R^2
**Urea**	$Y = -16.975X^2 + 51.92X + 79.468$	0.96
**Detergent**	$Y = 33.997X + 63.959$	0.98
**Soap**	$Y = 13.81X^2 + 35.314X + 4.7468$	0.98
**Salt**	$Y = 226.28X^3 - 520.93X^2 + 405.46X + 78.141$	0.99
**H_2O_2**	$Y = 50.489X^{0.4333}$	0.97
**$NaHCO_3$**	$Y = 97.605X^{0.1728}$	0.98
**Starch**	$Y = 12.105X^2 + 103.09X + 92.13$	0.98

S5. Color interference

The experimental images of the color interference study is shown in Fig. S2.

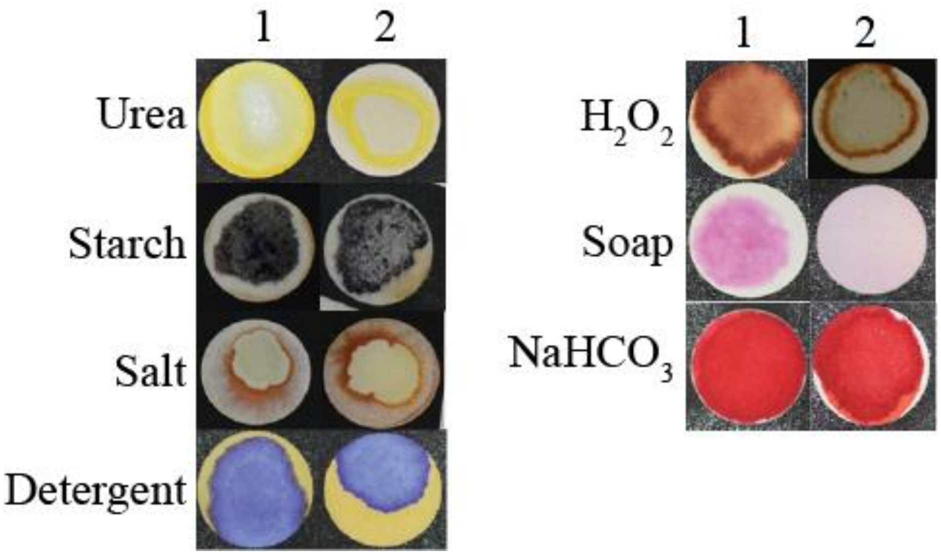

Figure S2: Single adulterants and mixture of adulterants represents by 1 and 2, respectively. Both the sample were made in a same volume of milk and tested for the colorimetric reaction. As we can see reagents are able to detect the particular adulterant from the mixture, we can easily identify the adulterants qualitatively. For quantitative detection we have showed the color intensity in the manuscript.

S6. The device

A prototype of a milk adulteration testing device is shown in Fig. S3. The name of the device is Milkit. Here simultaneous detection of seven adulterants is performed and from the image it is easily understandable that the device can detect all the adulterants at once.

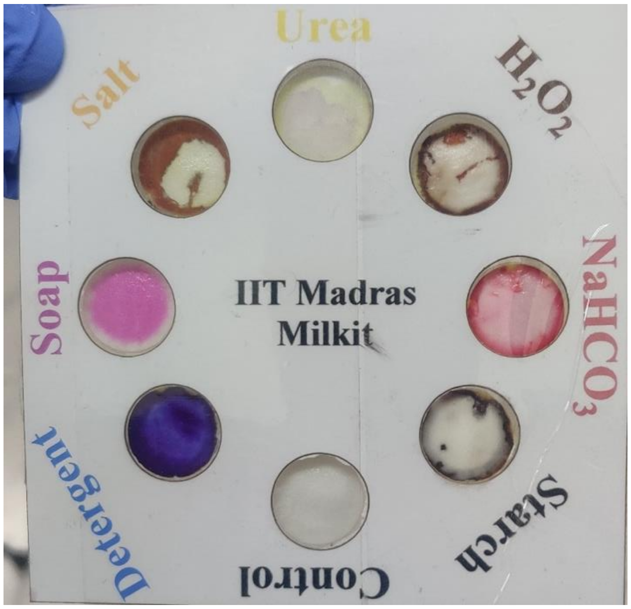

Figure S3: The handheld prototype of a device after use is shown here.

S7. Cost analysis

In this work we tried to develop a point of care device to test milk sample. The manufacturing cost of the device is 17 Rs. Where we can test 7 adulterants. The details price of the different ingredients of the device is listed in Table S4.

Table S4: Detail cost analysis for making a device.

Items	Cost (Rs.)
**Whatman filter paper grade 4**	13
**Double sided tape**	2.4
**Covers and PVC sheet**	2
**Reagent for urea**	0.002
**Reagent for H_2O_2**	0.02
**Reagent for starch**	0.02
**Reagent for salt**	0.15
**Reagent for soap**	0.01
**Reagent for detergent**	0.002
**Reagent for $NaHCO_3$**	0.06

S8. Comparison with the traditional methods

Table S5: Comparison with the traditional technique.

Methods	Adulterants	LOD	Time	Ref
FTIR	Urea, H_2O_2<br><br>Urea, H_2O_2, $NaHCO_3$, Salt, Starch	10 mg/dL, 0.014g/L<br><br>126 ppm, 200 ppm, 300ppm, >1%, 0.4%		1, 2<br><br>3

LC-TMS	Urea<br><br>Detergent	9 mg/dL<br><br>0.01% (w/w)	1.2 min<br><br>20min	4<br><br>5
Electrical impedance spectroscopy	H_2O_2, Soap<br><br>Soap	1% v/v, 1%v/v<br><br>0.1% w/w		6<br><br>7
NIR-RS	Urea	10 mg/dL		8
SERS	Urea	5 mg/dL		9
Fluorimetry	H_2O_2	90 µg/kg		10
Amperometry	H_2O_2	94 µg/kg	1 s	11
Electrical conductance	Salt, $NaHCO_3$<br><br>Soap, Detergent	0.09 g/L, 0.27 g/L<br><br>3ml/47ml, 0.5g/50ml	 <br>2-6 min	12<br><br>13
High-performance thin layer chromatography (HPTLC)	$NaHCO_3$	20mg/100ml		14
Mid-infrared spectroscopy	H_2O_2, $NaHCO_3$, Salt, Soap, Starch	21 g/L, 4 g/L, 5 g/L, 4 g/L, 5 g/L		15
This work	Urea, Starch, Salt, Detergent, H_2O_2, Soap, $NaHCO_3$	0.05%v/v, 0.1%v/v, 0.1%v/v, 0.2%v/v 0.1% v/v, 0.2% v/v, 0.2% v/v	30-40 Sec	

Where, FTIR= Fourier transform infrared spectroscopy, LC-TMS = liquid chromatography–tandem mass spectrometry, NIR-RS = Near-infrared Raman spectroscopy, SERS = surface enhanced Raman scattering.

A comparison among different traditional methods to detect milk adulteration is shown in Table S5. There are a few existing devices that are developed from the traditional methods for detecting milk adulteration in the market. Few examples of such devices are MilkoScan FT1 machine [16], Ecomilk analyzer [17], Ksheer sacnner [18], and Milk-o-test [19] which is developed by using FTIR, ultrasonic, electrochemical, and colorimetric techniques respectively. The cost of these devices varies from a few dollars to a million of dollars for colorimetric and FTIR techniques, respectively. From this study, it is clear that the cost of testing can be reduced by using the colorimetric technique, which can help the community to use the point-of-care device.

S9. Sample preparation

For testing pure milk sample and single adulterant, we have used the sample without any preparation step. However, for testing multiple adulterants simultaneously, we have added water in the milk after adding all the adulterants so that the lactometer shows the pure milk reading. In Fig. S4 it is easily visible that after adding all the adulterants in the milk sample the red mark (pure milk level) of the lactometer is increased because of the high density.

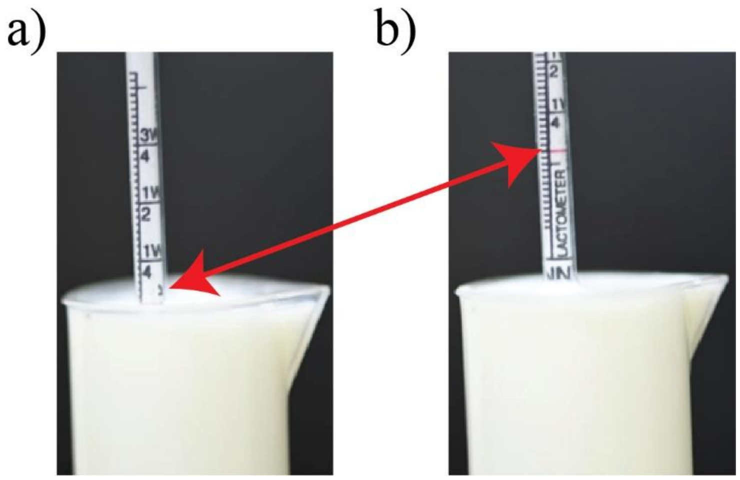

Figure S4: (a) Lactometer reading of pure milk. (b) Lactometer reading of adulterated milk. In the second case, the density of milk is increased due to the addition of adulterants. We have added extra water to the adulterated sample so that it again shows the pure milk reading. In figure b we can see the red mark which is the pure milk reading.

S10. Middle layer

The 3d paper device has 2 paper layers, one is transportation zone, and another is detection zone. Both the paper layers are connected by a supporting layer made by PVC sheet. Using glue, the paper layers are attached to the plastic layer. To make a less resistance to the flow we try to make the contact are of the paper and plastic layer less. So, we make some cuts in the plastic layer. The schematical view of the middle plastic layer is shown in Fig. S5.

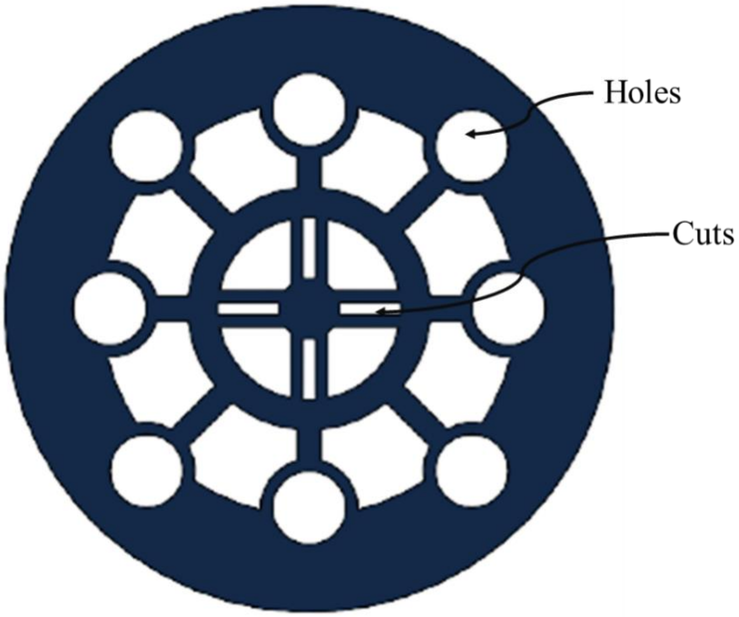

Figure S5: (a) Schematic of the plastic support. Where cuts are provided to decrease the resistance force and holes are provided to transfer the sample from the transportation zone to detection zone.

S11. Capillary rise

The liquid spreading is compared in Whatman Grade 1 and 4 filter paper. As the pore size is more in the Whatman Grade 4 filter paper the liquid spreading is also more. In Fig. S6 the distance cover by the liquid with time is shown for grade 1 and 4 filter paper.

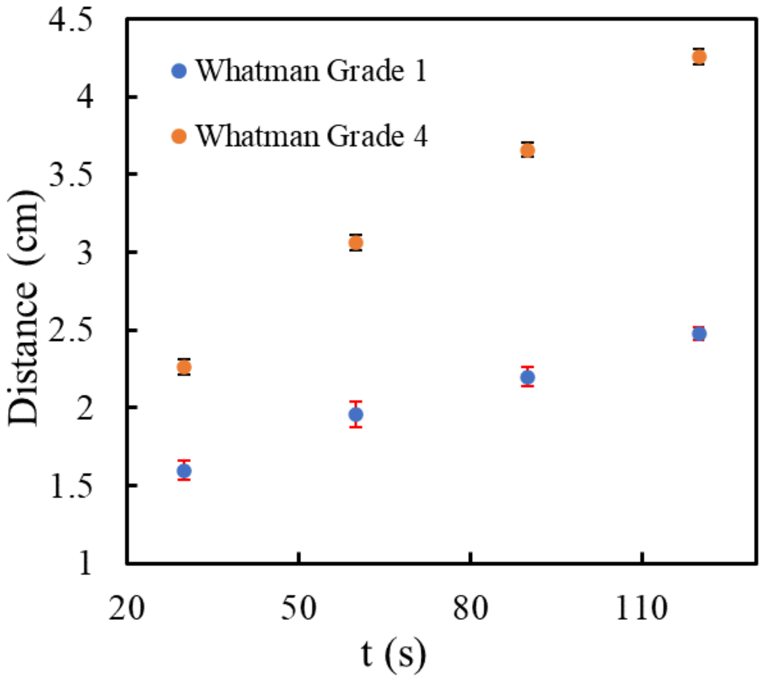

Figure S6: Comparison of distance covered by liquid sample in two different types of paper in a particular time interval. It is clear that in Whatman grade 4 filter paper the liquid flow is faster.

S12. Bayesian single-level linear regression model

In the manuscript we have showed the classical linear regression fit to form the calibration curves. We have showed a linear range for our data set using the linear fit and find out the sensitivity of the proposed method. Here we have performed the Bayesian linear regression fit for all the scattered data points of color intensity to find the linear variation of the color intensities with the adulterant's concentration. The Bayesian model is defined as $y_i \sim \text{Normal}(\beta_0 + \beta_1 x_i, \sigma^2)$, where the outcome is y_i, the predictor variable is x_i, β_0 is the intercept, coefficient is β_1 and σ^2 is the variance [20].

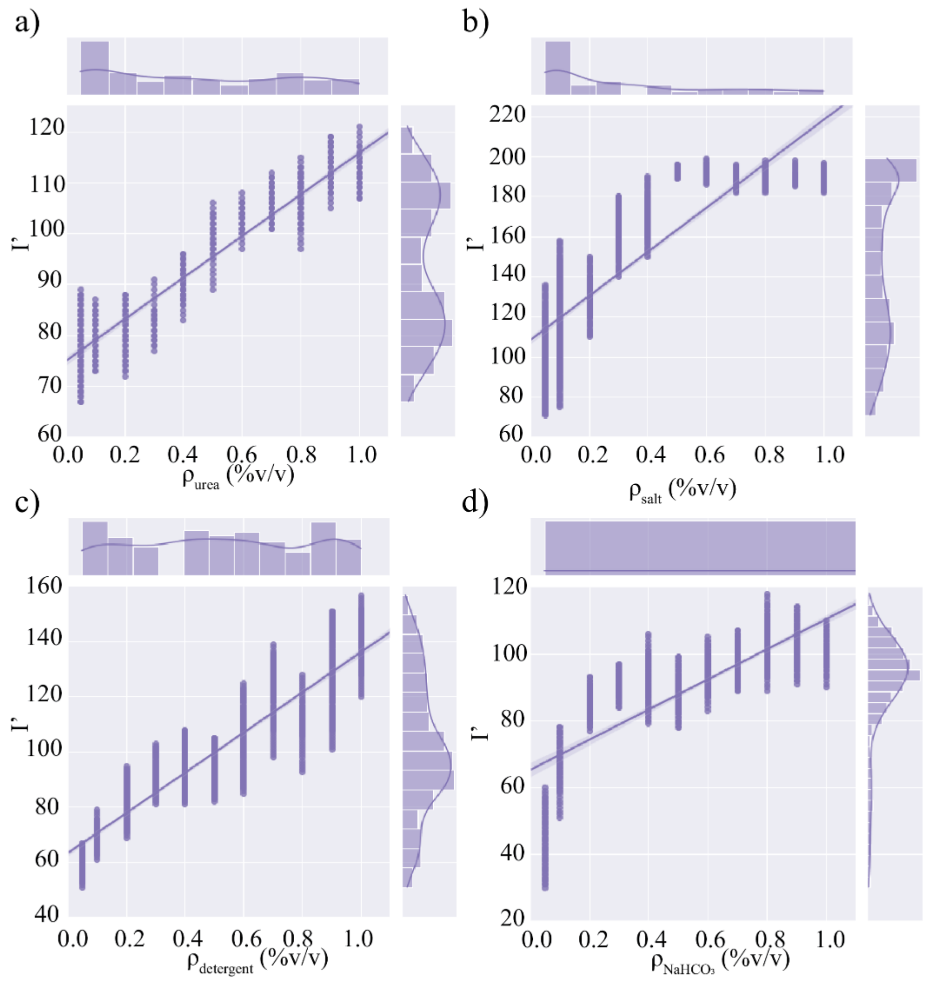

Figure S7: The scattered plot of the color intensity data vs concentration is shown here for different adulterants. Here we have considered all the data points of color intensity from the detection zone and a linear line is fitted with a 95% confidence interval is shown here.

We have used the Markov chain Monte Carlo (MCMC) based stan_glm function in rstanarm to fit Bayesian linear regression model. The summary of the Bayesian linear regression fit is listed in Table S6 where the mean value of intercepts, slope, and sigma (variance) are shown for 5%, 50%, and 95% confidence intervals. Rhat, n_eff, and sd are represented for the potential scale reduction factor on split chains, a crude measure of effective sample size, and standard deviation respectively. In Fig. S7 all the color intensity data corresponding to the adulterant's concentration is presented with a linear fitting line where the 95% confidence interval part is shown with shaded background.

Table S6: The details of the Bayesian linear fit are listed here for urea, salt, detergent, and sodium-bi-carbonate.

**Urea**							
**Parameters**	**Rhat**	**n_eff**	**mean**	**sd**	**5%**	**50%**	**95%**
**Intercept**	1.00	3478	75.6	0.4	74.88	75.6	76.26
**Slope**	1.00	3105	37.2	0.8	35.82	37.2	38.46
**Sigma**	1.00	2736	5.1	0.2	4.77	5.1	5.43
**Salt**							
**Parameters**	**Rhat**	**n_eff**	**mean**	**sd**	**5%**	**50%**	**95%**
**Intercept**	1	2337	38.7	2.9	33.82	38.7	43.50
**Slope**	1	2620	3.6	2.5	-0.57	3.6	7.65
**Sigma**	1	2156	110.3	3.9	104.07	110.3	116.96
**Detergent**							
**Parameters**	**Rhat**	**n_eff**	**mean**	**sd**	**5%**	**50%**	**95%**
**Intercept**	1	3273	69.0	0.8	67.71	69.0	70.20
**Slope**	1	2494	57.5	1.4	55.19	57.5	59.69
**Sigma**	1	2351	11.3	0.3	10.76	11.3	11.91
**$NaHCO_3$**							
**Parameters**	**Rhat**	**n_eff**	**mean**	**sd**	**5%**	**50%**	**95%**
**Intercept**	1	3258	68.4	0.8	67.15	68.4	69.70
**Slope**	1	3085	34.6	1.3	32.53	34.6	36.66
**Sigma**	1	3015	11.4	0.3	10.83	11.3	11.92

References

1) Shyam Narayan Jha et al. “Detection and quantification of urea in milk using attenuated total reflectance-Fourier transform infrared spectroscopy”. In: Food and Bioprocess Technology 8.4 (2015), 926–933.
2) PM Santos, ER Pereira-Filho, and LE Rodriguez-Saona. “Rapid detection and quantification of milk adulteration using infrared microspectroscopy and chemometrics analysis”. In: Food Chemistry 138.1 (2013), 19–24.

3) Per Waaben Hansen and Stephen E Holroyd. “Development and application of Fourier transform infrared spectroscopy for detection of milk adulteration in practice”. In: International Journal of Dairy Technology 72.3 (2019), 321–331.
4) Tanzina Azad and Shoeb Ahmed. “Common milk adulteration and their detection techniques”. In: International Journal of Food Contamination 3.1 (2016), 1–9.
5) Manjun Tay et al. “Rapid screening for detection and differentiation of detergent powder adulteration in infant milk formula by LC–MS”. In: Forensic Science International 232.1-3 (2013), 32–39.
6) Gabriel Durante et al. “Electrical impedance sensor for real-time detection of bovine milk adulteration”. In: IEEE Sensors Journal 16.4 (2015), 861–865.
7) Chirantan Das et al. “On-chip detection and quantification of soap as an adulterant in milk employing electrical impedance spectroscopy”. In: 2018 International Symposium on Devices, Circuits and Systems (ISDCS). IEEE. 2018, 1–4.
8) Khan Mohammad Khan et al. “Detection of urea adulteration in milk using near-infrared Raman spectroscopy”. In: Food Analytical Methods 8.1 (2015), 93–102.
9) Abid Hussain, Da-Wen Sun, and Hongbin Pu. “SERS detection of urea and ammonium sulfate adulterants in milk with coffee ring effect”. In: Food Additives & Contaminants: Part A 36.6 (2019), 851–862.
10) Chifang Peng, Chunli Liu, and Zhengjun Xie. “Preparation of a fluorescent silver nanoprism–dye complex for detection of hydrogen peroxide in milk”. In: Analytical Methods 7.23 (2015), 9749–9752.
11) Kavitha Thandavan et al. “Hydrogen peroxide biosensor utilizing a hybrid nano-interface of iron oxide nanoparticles and carbon nanotubes to assess the quality of milk”. In: Sensors and Actuators B: Chemical 215 (2015), 166–173.
12) Wesley William Gon ̧calves NASCIMENTO et al. “Results from portable and of low cost equipment developed for detection of milk adulterations”. In: Food Science and Technology 37 (2017), 38–41.
13) Moupali Chakraborty, Dina Anna John, and Karabi Biswas. “A statistical study of detergent and shampoo adulterated milk detection system”. In: 2016 IEEE Annual India Conference (INDICON). IEEE. 2016, 1–6.
14) Manish M Paradkar, Rekha S Singhal, and Pushpa R Kulkarni. “An approach to the detection of synthetic milk in dairy milk: 3. Detection of vegetable oil and sodium bicarbonate”. In: International Journal of Dairy Technology 54.1 (2001), 34–35.
15) Carina de Souza Gondim et al. “Detection of several common adulterants in raw milk by MID-infrared spectroscopy and one-class and multi-class multivariate strategies”. In: Food Chemistry 230 (2017), 68–75.
16) Foss Analytics. Milkoscan FT1 Machine. https://www.fossanalytics. com/en in/products/milkoscan-ft1.
17) Softrosys Technologies. Ecomilk Ultrasonic Milk Analyzers. https:// 5 . imimg . com / data5 / SELLER / Doc / 2020 / 9 / BR / NA / MG / 4338579 / ekomilk-ultra-milk-analyzer.pdf.
18) CSIR-Central Electronics Engineering Research Institute. Ksheer Scanner. https : / / www . indiamart . com / csirtech / products . html \#19712946848.
19) NDDB-National Dairy Development Board. Kit for Detection of Adulterants in Milk. https://www.nddb.coop/sites/default/files/pdfs/procurement/Technical\%20Specifications\%20of %20Milk\%20Adulteration\%20Detection\%20Kits.pdf.
20) Muth, C., Oravecz, Z. & Gabry, J. User-friendly Bayesian regression modeling: A tutorial with rstanarm and shinystan. Quant. methods for psychology 14, 99–119 (2018).
